# Protective effects of thiamine on *Wickerhamomyces anomalus* against ethanol stress

**DOI:** 10.3389/fmicb.2022.1057284

**Published:** 2022-12-07

**Authors:** Yinfeng Li, Hua Long, Guilan Jiang, Zhihai Yu, Mingzheng Huang, Shiping Zou, Tianbing Guan, Yan Zhao, Xiaozhu Liu

**Affiliations:** ^1^Guizhou Institute of Technology, Guiyang, China; ^2^Chongqing Key Laboratory of Industrial Fermentation Microorganism, Chongqing University of Science and Technology, Chongqing, China; ^3^College of Bioscience and Biotechnology, Hunan Agricultural University, Changsha, China

**Keywords:** ethanol stress, thiamine, *Wickerhamomyces anomalus*, transcriptomics, metabolomics

## Abstract

*Wickerhamomyces anomalus* (*W. anomalus*) is widely reported in the brewing industry and has positive effects on the aromatic profiles of wines because of its unique physiological characteristics and metabolic features. However, the accumulation of ethanol during fermentation inhibits the growth of *W. anomalus.* Thiamine is involved in the response against various abiotic stresses in microorganisms. Therefore, we used transcriptomic and metabolomic analyses to study the effect of thiamine on ethanol-stressed *W. anomalus*. The results indicate that thiamine could alleviate the inhibitory effect of ethanol stress on the survival of *W. anomalus.* Differentially expressed genes (DEGs) and differentially expressed metabolites (DEMs) caused by the thiamine intervention were identified as oxidative phosphorylation through integrated transcriptomic and metabolomic analyses. In addition, ethanol treatment decreased the content of intracellular adenosine triphosphate (ATP), while thiamine partially alleviated this phenomenon. The present comprehensive transcriptional overview and metabolomic analysis provide insights about the mechanisms of thiamine protection on *W. anomalus* under ethanol stress and promote the potential applications of *W. anomalus* in the fermentation industry.

## Introduction

The demand and consumption of ethanol have increased worldwide alongside the depletion of conventional energy resources and increased environmental pollution (Taghizadeh-Alisaraei et al., 2019). Yeast is commonly chosen as the main industrial ethanol producer for its efficient ethanol productivity (Adebami et al., 2022). However, yeast cells face ethanol stress during ethanol fermentation, especially with the increased ethanol concentration at the middle and late stages of fermentation (Saini et al., 2018). Ethanol inhibits the growth of yeast at a low concentration and reduces cell vitality and increases cell death at a high concentration in *Saccharomyces cerevisiae* (Jhariya et al., 2021). Ethanol also influences gene expression, signal transduction, and cell metabolism by regulating RNA and protein accumulation and altering metabolism (Morard et al., 2019).

Thiamine, also known as vitamin B_1_, is reported in many cellular physiological metabolic processes in the form of phosphorylation, such as the citric acid cycle (TCA cycle), acetyl-CoA production, pentose phosphate pathway, Calvin cycle, and synthesis of branched-chain amino acids (Goyer, 2010). Recent evidence demonstrates that thiamine also participates in the cell response against various stress (Rapala-Kozik et al., 2008). Thiamine functions as an activator of plant disease resistance and increases plant resistance to external environmental stress (Fitzpatrick and Chapman, 2020). Exogenous application of thiamine can attenuate high salt stress effect on plants and improve resistance to fungi, bacteria, and viruses in rice and *Arabidopsis* (Sayed and Gadallah, 2002; Ahn et al., 2005). Moreover, thiamine increased the resistance of baker’s yeast *S. cerevisiae* against oxidative, osmotic, and thermal stress through mechanisms partly independent of thiamine diphosphate-bound enzymes (Wolak et al., 2014). Moreover, thiamine stimulated yeast growth and affected the metabolism of *S. cerevisiae* during ethanol fermentation (Labuschagne and Divol, 2021).

Here, we report that thiamine has a protective effect on *Wickerhamomyces anomalus* against ethanol stress. Moreover, the underlying possible mechanism for the role of thiamine in ethanol tolerance was confirmed based on transcriptomic and metabolomic approaches.

## Materials and methods

### Yeast strains and culture conditions

The *W. anomalus* C11 strain was separated from the fruit spontaneous fermentation broth of *Rosa. roxburghii* Tratt (Liu et al., 2021) and cultured with yeast extract peptone dextrose medium (YEPD) at 28°C for 72 h and then kept at 4°C for later use.

### Ethanol stress conditions

Our previous research demonstrated that the strain of *W. anomalus* C11 could tolerate 9% (v/v) of ethanol, and 12% (v/v) ethanol had strong inhibiting effect on its growth. Here, we chose 12% (v/v) ethanol as treated concentration. Briefly, *W. anomalus* C11 was inoculated with 10^8^ cells/mL in YEPD broth medium and cultured at 28°C for 8 h to make the cell into logarithmic phase (Li et al., 2019; Liu et al., 2021). And the cultures were divided into a 0% ethanol group, a 12% ethanol group, and a 12% ethanol + thiamine group. The 0% ethanol group did not contain ethanol and was used as the control group. The yeast cells were treated with 12% (v/v) concentration of ethanol in the 12% ethanol group. In the 12% ethanol + thiamine group, yeast cells were treated with 12% (v/v) ethanol and 5 g/L of thiamine. Treatment began at 0 h. All groups were cultured for 6 h, and then, the cells were collected for the cell survival, transcriptomic, and metabolomic analyses.

### Spot analysis

After treatment, yeast cells from all groups were collected, washed twice with distilled water, and re-suspended in distilled water at the same cell concentration (OD_600_ = 1). The cell suspension was diluted to 10^0^, 10^–2^, and 10^–4^, and 2 μL of each diluent was spotted onto YEPD solid plates and incubated at 28°C for 36 h. Colonies were captured using a microscope (Olympus, Japan).

### Methylene blue staining

Death of yeast cells was checked by the methylene blue staining method of Sami et al. (1994). The dead cells were stained as blue, whereas the live cells were not stained. The % death cells were calculated by counting the number of dead cells in 10 random sights using a hemacytometer, and the average values were calculated.

### Biomass detection

Biomass was analyzed by measuring the dry weight of the cells of each group. Briefly, 30 mL of *W. anomalus* cells were collected by centrifugation at 4,000 × g for 10 min, followed by drying at 65°C until the weights were constant; mass was measured using an analytical balance (Lichen, China).

### Transcriptomics analysis

Total RNA was prepared from the yeast cells of each group using TRIzol reagent (Invitrogen, USA) according to the manufacturer’s instructions. Genomic DNA was digested using DNase I (TaKara, Japan). An RNA-seq transcriptome library was constructed with TruSeq™ RNA sample preparation kit from Illumina (San Diego, USA) using 1 μg of total RNA. Libraries were size selected for cDNA target fragments of 300 bp on 2% low range ultra agarose, followed by PCR amplification using Phusion DNA polymerase (NEB, USA) for 15 PCR cycles. The paired-end RNA-seq library was constructed with the Illumina HiSeqxten/NovaSeq 6000 sequencer (Illumina, USA).

The raw paired end reads were trimmed and quality controlled using SeqPrep^1^ and Sickle^2^ with default parameters. Then, clean reads were separately aligned to the reference genome with orientation mode using HISAT2^3^ software. The mapped reads of each sample were assembled by StringTie^4^ in a reference-based approach. To identify differentially expressed genes (DEGs), the expression level of each transcript was calculated according to the transcripts per million reads method. RSEM^5^ was used to quantify gene abundances.

In addition, KEGG pathway analysis was carried out with GOATOOLS^6^ and KOBAS.^7^

### Metabolomics analysis

Liquid chromatography-mass spectrometry (LC-MS)-based metabolomics was performed by Majorbio Biotech (Shanghai, China) to detect the differentially expressed metabolites (DEMs). Briefly, 50 mg of the samples of each group was accurately prepared, and the metabolites were extracted using 400 μL of methanol: water (4:1, v/v) solution containing 0.02 mg/mL of L-2-chlorophenylalanine as internal standard. The mixture was allowed to settle at –20°C, vortexed for 30 s, and ultrasonicated at 40 kHz for 30 min at 5°C. The supernatant was carefully prepared for LC-MS analysis after centrifugation at 13,000 × g for 15 min. The raw data were imported into Progenesis QI 2.3 (Waters, USA) for peak detection and alignment. The preprocessing results generated a data matrix that comprised retention time, mass-to-charge ratio values, and peak intensity. Annotating of the metabolites and differential metabolite analysis of each group were carried out on the Majorbio Cloud Platform.^8^

### Measurement of adenosine triphosphate levels

The content of the intracellular adenosine triphosphate (ATP) was measured using an ATP assay kit (Beyotime, Shanghai, China) according to the manufacturer’s instructions. The relative content of ATP was calculated with the value of the experimental group divided by the value of the control group.

### Statistics

The results are shown as the mean ± standard deviation (SD). Univariate analysis of variance (ANOVA) of the data and the significance of the difference test were performed using SPSS 21.0 software (IBM-SPSS Inc., Chicago, IL, USA). The statistical significance level was set at 5%.

## Results

### Thiamine alleviates the inhibitory effect of ethanol stress on the survival of *Wickerhamomyces anomalus*

**FIGURE 1:**
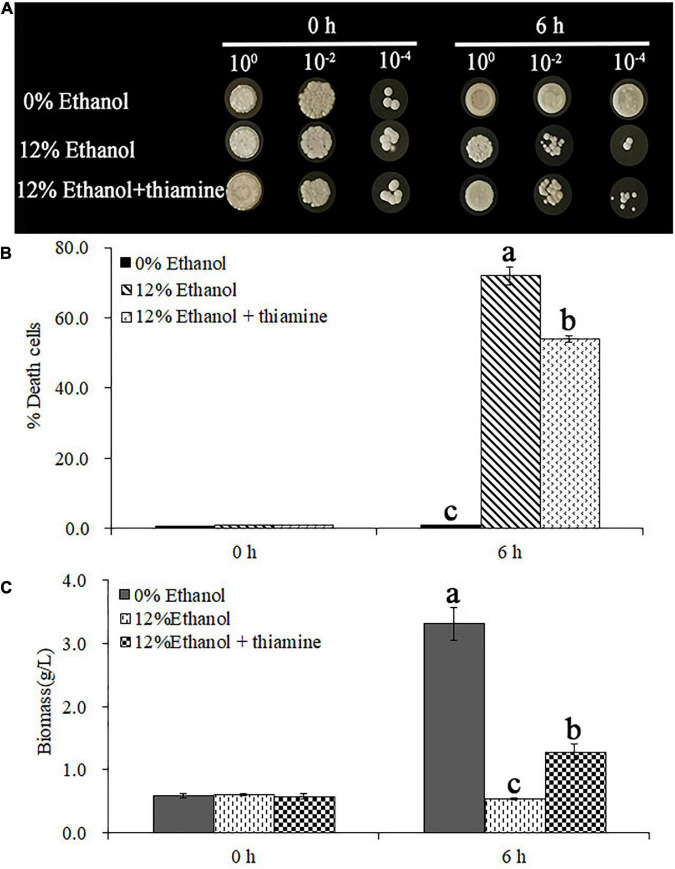
Thiamine improved the survival of *Wickerhamomyces anomalus* cells under ethanol stress. **(A)** Growth of yeast cells on yeast extract peptone dextrose medium (YEPD) solid media, as detected by spot dilution assay. **(B)** Death rate of yeast cells from different treatment groups. **(C)** Biomass of yeast cells from different treatment groups.

As shown in Figures 1A,B, the survival of yeast cells was strongly inhibited by treatment with 12% ethanol; most of the cells were dead. In contrast, thiamine effectively enhanced the survival of the yeast cells and significantly decreased the% death cells. In addition, the reduced biomass caused by ethanol stress was partially reversed by exogenous thiamine supplementation (Figure 1C).

### Identification of differentially expressed genes by transcriptomics

In total, 47,294,837.33, 44,135,694.67, and 48,045,852.67 raw reads were obtained in the 0% ethanol, 12% ethanol, and 12% ethanol + thiamine treatment groups, respectively, and 46 859 291.33, 43 675 678, and 47 486 968.67 clean reads were obtained from these three groups, respectively. The error rate of all the groups was 0.02%, and the values of Q20 (%) and Q30 (%) were greater than 90% (Table 1). Moreover, principal component analysis (PCA) results indicate that ethanol-treated samples were well separated from the control group (Supplementary Figure 1), which indicates that the quality of sequencing samples was acceptable and the data of this transcriptome sequencing were accurate.

**TABLE 1:** Quality analysis of transcriptome sequencing data of different groups.

Group	Raw reads	Raw bases	Clean reads	Clean bases	Error rate (%)	Q20 (%)	Q30 (%)	GC (%)
0% ethanol	47,294,837.33	7,141,520,437	46,859,291.33	6,969,933,135	0.02	98.75	95.84	38.65
12% ethanol	44,135,694.67	6,664,489,895	43,675,678	6,501,374,566	0.02	98.12	94.13	38.19
12% ethanol + thiamine	48,045,852.67	7,254,923,753	47,486,968.67	7,043,020,903	0.02	98.46	95.07	38.19

### Defining differentially expressed genes

To further probe the DEGs induced by thiamine, the screening criteria were set to *P* < 0.05 and | log2 (FC) | > 2. It was found that 2,274 DEGs were induced by 12% ethanol treatment, of which 1,153 DEGs were upregulated and 1,121 DEGs were downregulated (Supplementary Figure 2A and Supplementary Table 1). There were 347 DEGs, of which 204 DEGs were upregulated and 143 DEGs were downregulated, detected after addition of thiamine (Supplementary Figures 2B,C and Supplementary Table 2).

### Gene ontology classification analysis of differentially expressed genes

**FIGURE 2:**
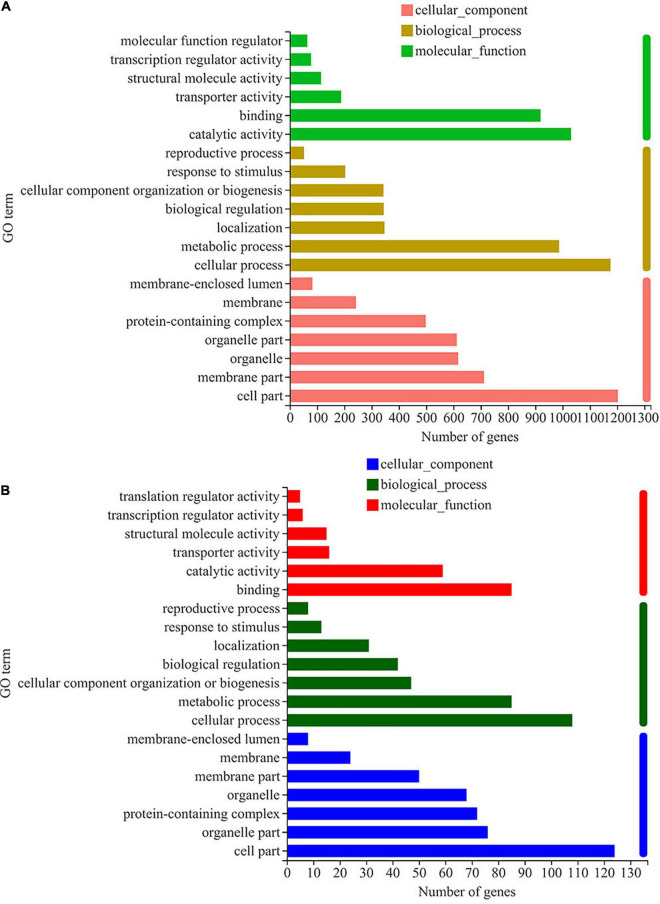
Gene ontology classification analysis of differentially expressed genes in *W. anomalus* after thiamine intervention under ethanol stress. **(A)** Gene ontology (GO) classification analysis of differentially expressed genes (DEGs) between 12% ethanol treatment group and 0% ethanol treatment group. **(B)** GO classification analysis of DEGs between 12% ethanol + thiamine treatment group and 12% ethanol treatment group.

To further explore the functional categories of DEGs after thiamine intervention under ethanol stress, they were categorized into 20 functional sub-categories by gene ontology (GO) classification analysis. As shown in Figure 2A, the DEGs induced by ethanol stress in *W. anomalus* were distributed in the GO terms of cellular component, biological process, and molecular function. In the cellular component category, the DEGs were mainly distributed in the term “cell part.” The GO terms enriched in the biological process category were “cellular process” and “metabolic process.” For the molecular function category, most DEGs were distributed in “catalytic activity” and “binding.” These data indicate that ethanol destroyed the cells’ structures and disturbed the intracellular metabolic reactions of *W. anomalus*.

It should be noted that the number of DEGs distributed in these GO terms, including “cell part,” “cellular process,” “metabolic process,” “catalytic activity,” and “binding,” decreased dramatically when thiamine was applied to *W. anomalus* under ethanol stress (Figure 2B). Therefore, thiamine could reverse these abnormally expressed genes induced by ethanol stress.

### KEGG pathway analysis of differentially expressed genes

**FIGURE 3:**
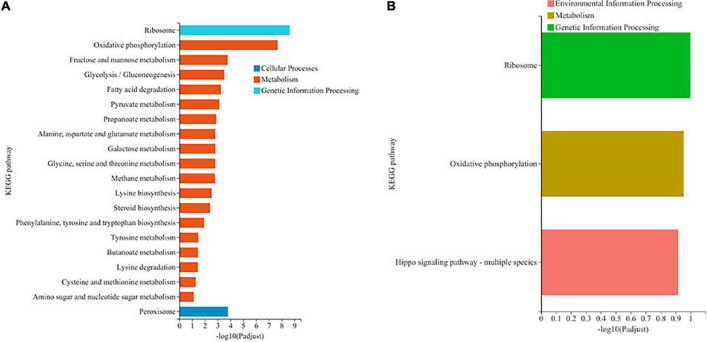
KEGG pathway analysis of differentially expressed genes (DEGs) in *W. anomalus* after thiamine intervention under ethanol stress. **(A)** KEGG pathway analysis of DEGs between 12% ethanol treatment group and 0% ethanol treatment group. **(B)** KEGG pathway analysis of DEGs between 12% ethanol + thiamine treatment group and 12% ethanol treatment group.

KEGG pathway analysis of DEGs was carried out, and the results are shown in Figure 3. Most the DEGs regulated by ethanol stress were annotated to “ribosome,” followed by “oxidative phosphorylation,” which suggest that ethanol stress disturbed intracellular protein synthesis and energy metabolism (Figure 3A). In addition, peroxisome was also annotated indicating peroxisome was involved in response to ethanol stress in *W. anomalus*, which were also found in *S. cerevisiae* (Teixeira et al., 2009). After thiamine intervention, the results of KEGG pathway analysis showed that the “ribosome,” “oxidative phosphorylation,” and “Hippo signaling pathway” were annotated, which indicate their critical role in response to ethanol stress (Figure 3B).

### Identification of differentially expressed metabolites by metabolomics

PCA was performed to check the quality of the metabolome sequencing samples. The results demonstrate that the sequencing samples of each group in the positive and negative ion modes were in a confidence circle, which was separated from others (Supplementary Figure 3).

### Defining differentially expressed metabolites

DEMs were first identified with the following criteria: variable importance in the projection (VIP) > 1 and *P* < 0.05. Under 12% ethanol treatment, there were 4,409 ion peaks and 296 annotated DEMs, including 146 upregulated DEMs and 150 downregulated DEMs (Supplementary Figure 4A and Supplementary Table 3). After thiamine intervention, 3,813 ion peaks and 321 annotated DEMs were detected in both the positive ion and negative ion modes, of which 63 DEMs were upregulated and 258 DEMs were downregulated (Supplementary Figure 4B and Supplementary Table 4).

### KEGG compound classification analysis of differentially expressed metabolites

**FIGURE 4:**
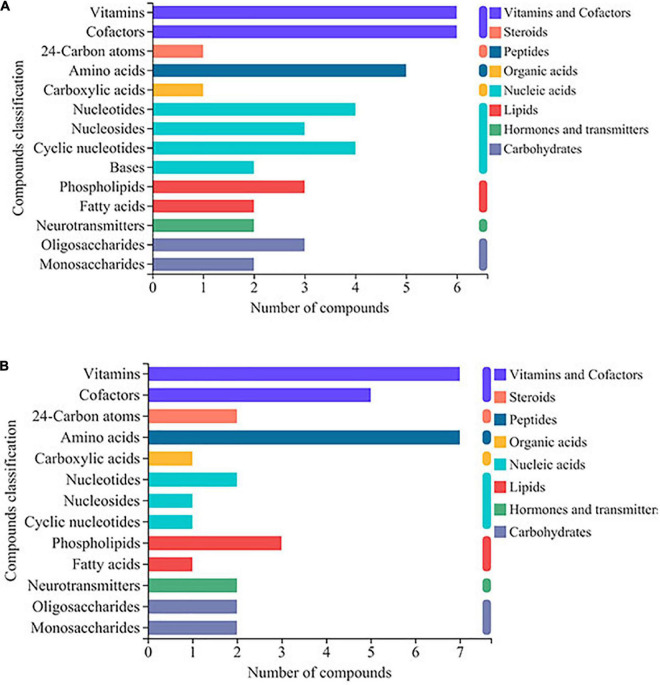
KEGG compound classification analysis of differentially expressed metabolites (DEMs) in *W. anomalus* after thiamine intervention under ethanol stress. **(A)** KEGG compound classification analysis of DEMs between 12% ethanol treatment group and 0% ethanol treatment group. **(B)** KEGG compound classification analysis of DEMs between 12% ethanol + thiamine treatment group and 12% ethanol treatment group.

DEMs were further identified using the KEGG compound database. The DEMs were categorized as vitamins and cofactors, steroids, peptides, organic acids, nucleic acids, lipids, hormones and transmitters, and carbohydrates. For the DEMs regulated by ethanol stress, vitamins, and cofactors were the largest identified compounds, followed by amino acids and then nucleotides and nucleosides (Figure 4A). After thiamine addition, many amino acid-related DEMs increased; in contrast, many compounds related to cofactors, nucleic acids, fatty acids, and oligosaccharides decreased (Figure 4B).

### KEGG pathway enrichment analysis of differentially expressed metabolites

KEGG pathway enrichment analysis was conducted to further probe the functions of these DEMs after thiamine intervention. The DEMs induced by ethanol treatment were significantly enriched in pantothenate and CoA biosynthesis, arginine biosynthesis, ABC transporters, purine metabolism, glutathione metabolism, and pyrimidine metabolism (Supplementary Figure 5A). After thiamine intervention, the enriched pathways were related to cysteine and methionine metabolism, glutathione metabolism, pantothenate and CoA biosynthesis, ABC transporters, beta-alanine metabolism, arginine biosynthesis, riboflavin metabolism, and pyrimidine metabolism (Supplementary Figure 5B).

### Integrated transcriptomics and metabolomics analysis

**FIGURE 5:**
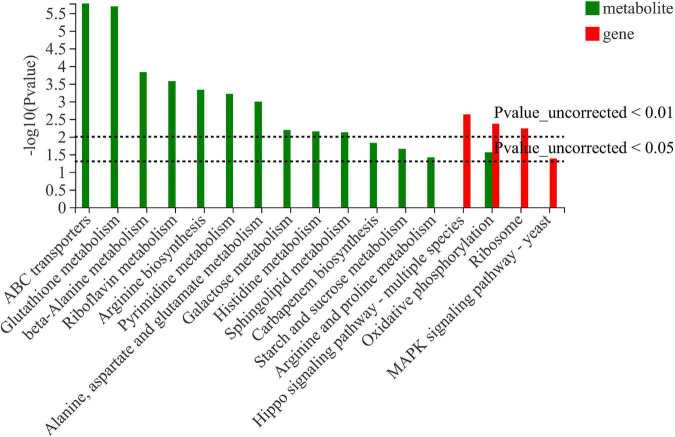
Integrated analysis of differentially expressed genes and differentially expressed metabolites in *W. anomalus* after thiamine intervention under ethanol stress.

Integrated analysis was carried out to further examine the data received from transcriptomics and metabolomics. As shown in Figure 5, oxidative phosphorylation was confirmed both in transcriptomics and metabolomics sequencing, suggesting that thiamine intervention alleviates the inhibitory effect on the energy metabolism by ethanol stress.

### Determination of adenosine triphosphate

**FIGURE 6:**
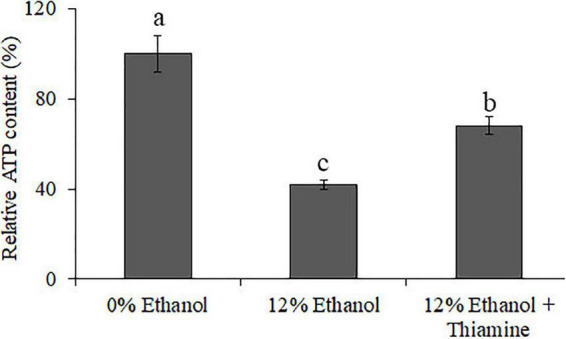
Relative content of intracellular ATP in *W. anomalus* after thiamine intervention under ethanol stress.

The results of ATP measurement (Figure 6) show that the relative content of ATP was significantly lower in the 12% ethanol group compared to control group, indicating that ethanol treatment can lead to a decrease in energy generation. The relative content of ATP of the “12% ethanol + thiamine group” was significantly higher than that of the 12% ethanol group but lower than that of the 0% ethanol group. The above results indicate that ethanol treatment decreased the content of intracellular ATP, while thiamine intervention partially alleviated this phenomenon.

## Discussion

In recent years, *W. anomalus* has received wide attention, and an increasing number of strains are being used in wine production because of its unique physiological characteristics and metabolic features (Padilla et al., 2018). More variety and greater quantities of aromatic esters and higher alcohols are detected when apple cider is co-fermented with *W. anomalus* and *S. cerevisiae* (Ye et al., 2014). In addition, the co-culture of *W. anomalus* and *S. cerevisiae* had a positive effect on ethyl acetate production and provides opportunities for altering the aroma and flavor perception of baijiu (Fan et al., 2019). A wild-type *W. anomalus* strain that produced 2-phenylethanol was obtained from rice wine by Tian et al. (2020), and the content of 2-phenylethanol reached 4,727.3 mg/L under optimal conditions. However, the mechanism underlying *W. anomalus* cells’ response to ethanol stress during ethanol fermentation has not been fully elucidated, which greatly limits its application in the production of ethanol beverages. Here, we found that ethanol stress has pleiotropic effects on *W. anomalus* cells, including inhibiting the survival of yeast cells (Figure 1), destroying the structure of cells (Figure 2), and disturbing the amino acid and energy metabolisms (Figures 3, 5, 6).

Much evidence indicates that thiamine is involved in the response against various abiotic stresses in plants (Li et al., 2022), animals (Chauhan et al., 2018), and microorganisms (Zhu et al., 2021). Studies show that the addition of a certain amount of thiamine affected the physiological and metabolic processes of *Zygosaccharomyces rouxii*, so that the strain could faster and better adapt to the high-salt environment and increase the production of the aromatic substance phenylethanol (Yao et al., 2020). For the *S. cerevisiae* cells under oxidative stress conditions, thiamine could maintain the redox balance partly independent of the functions of TDP-dependent enzymes (Wolak et al., 2014). Thiamine also stimulated yeast growth and promoted the metabolism of ethanol in *S. cerevisiae* during ethanol fermentation (Labuschagne and Divol, 2021). In addition, thiamine was reported had protective effects in *Candida* spp. against oxidative stress (Wolak et al., 2015). Here, we found that thiamine alleviated the inhibitory effect of ethanol stress on the *W. anomalus* cells probably by improving energy metabolism (Figures 5, 6), and the underlying mechanism of thiamine mediation will be further explored in the future.

It is generally believed that thiamine mainly alleviates the dysregulation of energy metabolism caused by various stresses in the form of phosphorylation (Goyer, 2010). Therefore, the relative content of ATP of the “12% ethanol + thiamine group” was significantly higher than that of the 12% ethanol group (Figure 6). Furthermore, the contents of many metabolites, such as amino acid, fatty acids, nucleic acids, and oligosaccharides were also changed with the exogenous supplementation of thiamine. For example, metabolite profiling highlighted the increased abundance of amino acid in thiamine-added *Lactococcus lacti*s cells during acid stress (Zhu et al., 2021). In this study, we also found that the contents of many amino acids, nucleic acids, fatty acids, and oligosaccharides-related DEMs increased or decreased after thiamine addition in *W. anomalus* (Figure 4B). The connection between thiamine metabolism and these aboved metabolites will be further explored in our next research.

The majority of yeast intracellular thiamine is thiamine pyrophosphate (TPP), and fulfills a fundamental function as a cofactor for several enzymes involved in energy metabolism pathways in eukaryotes mainly involves glycolysis, TCA cycle, and oxidative phosphorylation occurring in mitochondria (Malecki et al., 2016). It was reported that ethanol induces reactive oxygen species (ROS) burst and autophagy because of the dysfunctional mitochondria in *S. cerevisiae* (Jing et al., 2018). Research also demonstrated that TPP-dependent Transketolase activity is involved in the cell’s oxidative stress response (Labuschagne and Divol, 2021). In the present study, we found an improved effect of thiamine on the oxidative phosphorylation and ATP production in *W. anomalus* under ethanol stress (Figures 3, 6), suggesting that the thiamine-regulated target is mitochondria. Therefore, the ROS content, mitochondrial structure, and the activity of Transketolase will be further studied in our future experiments.

The Hippo pathway has emerged as a conserved signaling pathway that is essential for the proper regulation of organ growth in *Drosophila* and vertebrates (Rock et al., 2013). In yeast, the Hippo pathway was also found, and it can be activated by phosphorylation-dependent assembly of signaling complexes (Halder and Johnson, 2011). However, the number of reports on the roles of the Hippo pathway in yeast remain few. Here, the Hippo signaling pathway was annotated through KEGG pathway analysis of DEGs (Figure 3B), which suggests that the Hippo pathway may be involved in the process of responding to ethanol stress in *W. anomalus*.

## Conclusion

To the best of our knowledge, this study is the first to systematically analyze the protective effects of thiamine on *W. anomalus* against ethanol stress based on transcriptomic and metabolomic approaches. Thiamine alleviated the inhibitory effect of ethanol stress on the survival of *W. anomalus* probably through improving the energy metabolism in mitochondria. The present comprehensive transcriptional overview and metabolomic analysis provide insights about the mechanisms underlying the improved effect of thiamine on *W. anomalus* against ethanol stress and promote the potential applications of *W. anomalus* in the fermentation industry.

## Data availability statement

The datasets presented in this study can be found online at https://www.ncbi.nlm.nih.gov/, accession number PRJNA908640.

## Author contributions

YL and XL wrote the original draft manuscript. HL and GJ conducted the experiments. ZY, MH, SZ, TG, and YZ helped to analyze the data of transcriptomics and metabolomics. XL conceived and designed the experiments. All authors contributed to the article and approved the submitted version.

## Footnotes

1
https://github.com/jstjohn/SeqPrep

2
https://github.com/najoshi/sickle

3
http://ccb.jhu.edu/software/hisat2/index.shtml

4
https://ccb.jhu.edu/software/stringtie/index.shtml?t=example

5
http://deweylab.biostat.wisc.edu/rsem/

6
https://github.com/tanghaibao/Goatools

7
http://kobas.cbi.pku.edu.cn/home.do

8
https://cloud.majorbio.com
