# Supplementary material for: Protective effects of thiamine on Wickerhamomyces anomalus against ethanol stress
Source: Front Microbiol. 2022 Dec 7;13:1057284. doi: 10.3389/fmicb.2022.1057284 (PMC9769406; doi:10.3389/fmicb.2022.1057284)

Supplementary Figures

Figure S1. Results of principal component analysis(PCA) of the samples for transcriptomics.


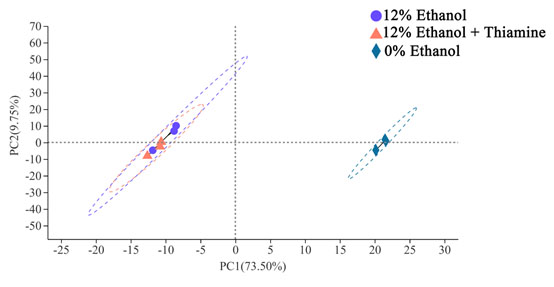


Figure S2. Differentially expressed genes (DEGs) in *W. anomalus* induced by thiamine addition. A. Results of DEGs shown in the volcano plot between 12% ethanol treatment group and control group. B. Results of DEGs shown in the volcano plot between 12% ethanol + thiamine group and 12% ethanol treatment group. C. Results of DEGs of different groups shown by pie chart.


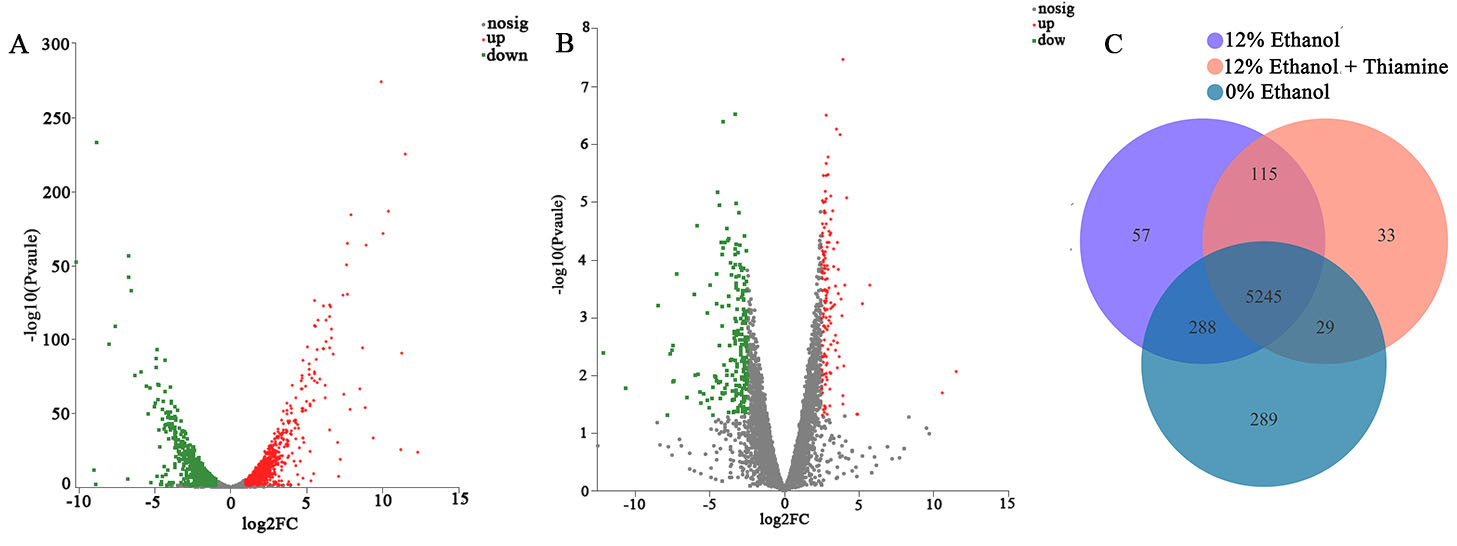


Figure S3. Results of principal component analysis(PCA) of the samples for transcriptome metabonomics. A, Positive ion mode; B, Negative ion mode.


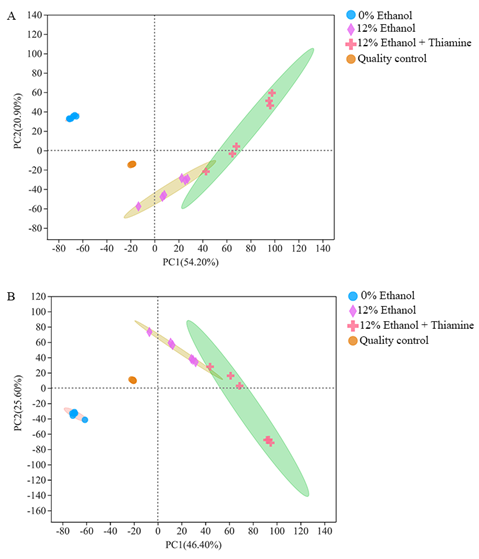


Figure S4 Differentially expressed metabolites (DEMs) in *W. anomalus* after thiamine intervention under ethanol stress. A. Results of DEMs shown in volcano plot between 12% ethanol treatment group and control group. B. Results of DEMs shown in volcano plot between 12% ethanol + thiamine treatment group and 12% ethanol treatment group.


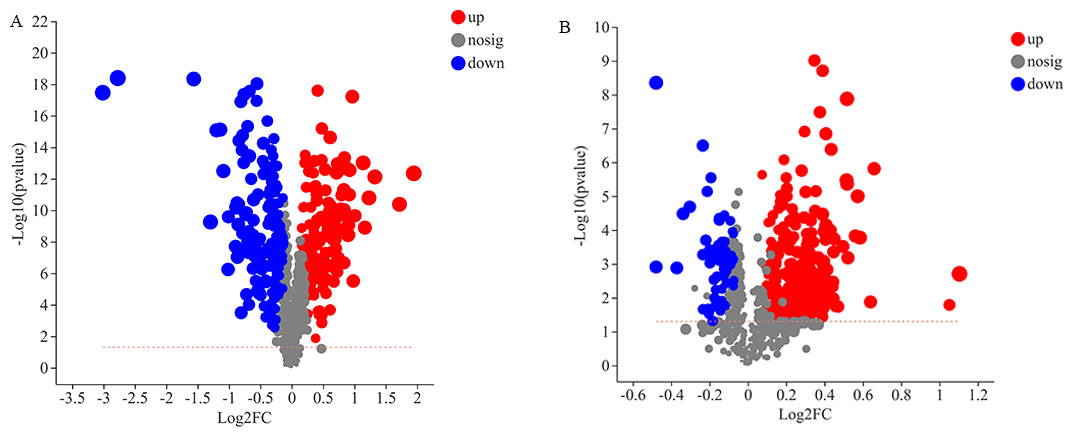


Figure S5 KEGG pathway enrichment analysis of DEGs in *W. anomalus* after thiamine intervention under ethanol stress. A. KEGG pathway enrichment analysis of DEGs between 12% ethanol treatment group and 0% ethanol treatment group. B. KEGG pathway enrichment analysis of DEGs between 12% ethanol + thiamine treatment group and 12% ethanol treatment group.


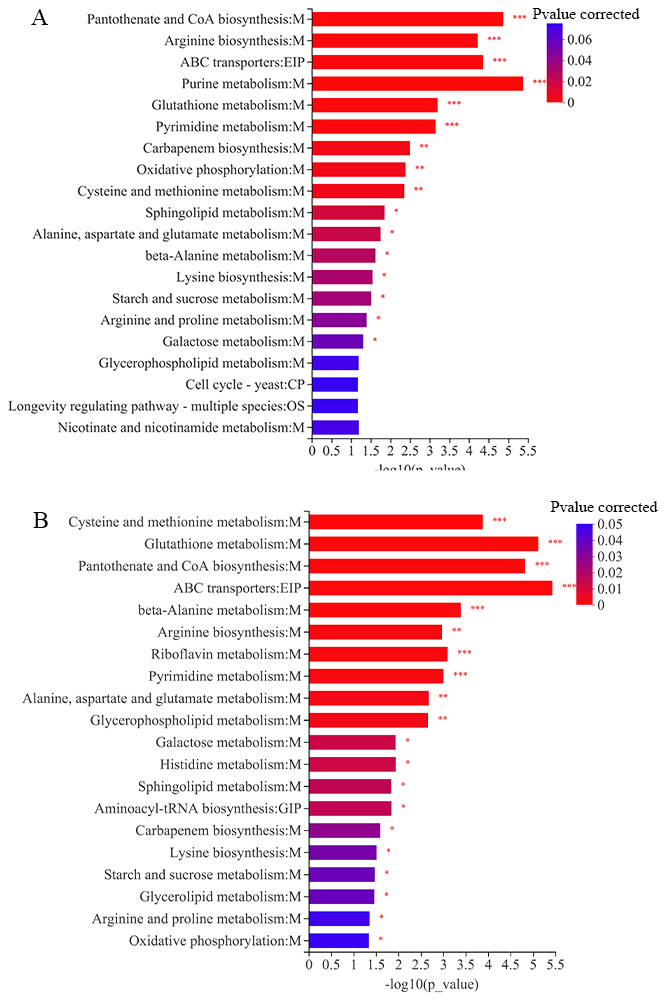

Supplement: Supplementary file 1 [file Data_Sheet_1.DOCX]
